# Prevention of Metabolic Impairment by Dietary Nitrate in Overweight Male Mice Improves Stroke Outcome

**DOI:** 10.3390/nu17152434

**Published:** 2025-07-25

**Authors:** Ellen Vercalsteren, Dimitra Karampatsi, Carolina Buizza, Gesine Paul, Jon O. Lundberg, Thomas Nyström, Vladimer Darsalia, Cesare Patrone

**Affiliations:** 1 Department of Clinical Science and Education, Internal Medicine, Karolinska Institutet, Södersjukhuset, 118 83 Stockholm, Sweden; dimitra.karampatsi@gmail.com (D.K.); thomas.nystrom@ki.se (T.N.); vladimer.darsalia@ki.se (V.D.); 2 Department of Clinical Science, Translational Neurology Group and Wallenberg Center for Molecular Medicine, Lund University, 221 84 Lund, Sweden; carolina.buizza@med.lu.se (C.B.); gesine.paul-visse@med.lu.se (G.P.); 3 Department of Pharmacology and Physiology, Karolinska Institutet, 171 56 Stockholm, Sweden; jon.lundberg@ki.se

**Keywords:** stroke, obesity, diabetes, nitrate, overweight

## Abstract

**Background/objectives**: Being overweight increases the predisposition to obesity and type 2 diabetes (T2D), which significantly elevate stroke risk and the likelihood of severe post-stroke disability. Dietary nitrate (NO_3_^−^) supplementation can mitigate obesity and metabolic impairments, making it a promising approach to halt overweight people from developing overt obesity/T2D, thereby potentially also improving stroke outcome. We determined whether NO3^−^ supplementation prevents overweight mice from progressing into obesity and T2D and whether this intervention improves stroke outcome. **Methods**: An overweight condition was induced via 6 weeks of a high-fat diet (HFD), after which animals were randomized to either a HFD or a HFD with NO_3_^−^ supplementation. After 24 weeks, when HFD-mice without NO_3_^−^ developed obesity and T2D, all animals were subjected to transient middle cerebral artery occlusion and stroke outcome was assessed via behavioral testing and infarct size. The effect of NO_3_^−^ on post-stroke neuroinflammation, neurogenesis, and neovascularization was analyzed by immunohistochemistry. **Results**: Sustained NO_3_^−^ supplementation in overweight mice did not prevent obesity or insulin resistance. However, it attenuated weight gain, prevented hyperglycemia, and significantly improved functional recovery after stroke, without affecting infarct size. Moreover, NO_3_^−^ decreased post-stroke neuroinflammation by reducing microglial infiltration. NO_3_^−^ did not affect stroke-induced neurogenesis or vascularization. **Conclusion**: These results highlight the potential of NO_3_^−^ supplementation to prevent metabolic impairment in the overweight population and improve stroke prognosis in this large group of people at risk of stroke and severe stroke sequelae.

## 1. Introduction

The worldwide epidemic of obesity has dramatically increased the incidence of metabolic syndrome, type 2 diabetes (T2D), and related cardiovascular complications [1,2,3,4]. One of the major debilitating consequences of this is stroke, which is a major cause of adult disability worldwide [5,6]. Therefore, there is a large global medical need to reduce obesity and T2D, to consequently also reduce stroke risk and poor stroke outcome.

Weight loss has proven effective in reducing the risk for cardiovascular disease in the obese population, primarily via its effective prevention of weight-related co-morbidities such as T2D [7,8,9,10]. In recent years, effective pharmacological strategies, such as high-dose glucagon-like peptide (GLP) 1 receptor agonists and dual GLP1R/glucose-dependent insulinotropic polypeptide (GIP) agonists, have been approved for weight loss [11,12,13]. However, current clinical guidelines only recommend the use of these drugs in individuals with obesity or who are overweight and have a diagnosed co-morbidity. Since most people who are overweight have not yet developed weight-related co-morbidities [14,15], this leaves a large population at an increased risk for stroke and poor stroke outcome with very limited options for intervention. Indeed, the only available strategy for these people are lifestyle changes (i.e., diet and exercise) that often face major problems with adherence [16,17]. In summary, sustainable efficacious interventions specifically targeting the overweight population in the preventive perspective to halt obesity/T2D and reduce stroke risk and stroke-induced disability are lacking.

Dietary nitrate (NO_3_^−^) is a novel strategy that effectively slows weight gain in obesity and T2D [18,19,20,21], making it a promising and feasible approach to evade obesity and T2D in the overweight population, thereby potentially also reducing stroke risk and persistent post-stroke disability. Nitrate is found in particularly high levels in green leafy vegetables such as lettuce and spinach and in beetroot [22]. Upon ingestion, NO_3_^−^ is metabolized via the nitrate–nitrite–NO pathway, which elicits a range of beneficial effects. For instance, NO activates AMP-activated protein kinase (AMPK), which inhibits fatty acid synthesis, promotes fatty acid oxidation, and enhances glucose uptake [22,23]. Additionally, NO_3_^−^ has been shown to improve insulin sensitivity and endothelial function, and reduce oxidative stress, underscoring its multifaceted role in metabolic health [22,24]. NO_3_^−^ also exerts direct beneficial effects on brain vascular health [25,26,27]. Moreover, dietary nitrate effectively reduces stroke risk [28,29], and improves stroke outcome in metabolically healthy rodents [30,31,32], potentially via its blood pressure-lowering effects [33,34] or beneficial effects on neuroinflammation [35], processes that are closely linked with stroke outcome. However, whether halting obesity and metabolic impairments by dietary NO_3_^−^ in the overweight population improves stroke outcome has not yet been investigated.

Therefore, the aim of this study was to determine whether prolonged NO_3_^−^ supplementation prevents the progression of the overweight condition into obesity and T2D and consequently improves stroke outcome in a clinically relevant murine model. Moreover, the effects of prolonged NO_3_^−^ supplementation on post-stroke neuroinflammation, neurogenesis, and neovascularization were assessed.

## 2. Materials and Methods

### 2.1. Animals

Twenty C57BL/6JRj mice (Janvier Labs, Le Genest-Saint-Isle, France) were used in this study. The mice were housed in environmentally controlled conditions (22 ± 0.5 °C, 12/12 h light/dark cycle with ad libitum access to food and water). The mice were kept in pathogen-free conditions in type III-size individually ventilated cages with wood chip bedding and nest material.

### 2.2. Sample Size Calculation

Group sizes were determined based on ≈20% effect size between the groups in functional recovery after stroke, with α = 0.05 and a statistical power of 90%. The standard deviation (SD) used in sample size calculation was obtained from pilot experiments. Based on this analysis, a minimum final sample size of *n* = 5 was determined. Taking into consideration the success rate of stroke surgery, mortality rate, and the likelihood of statistical outliers, the final sample size per group was set at *n* = 10.

### 2.3. Experimental Design

**Figure 1:**
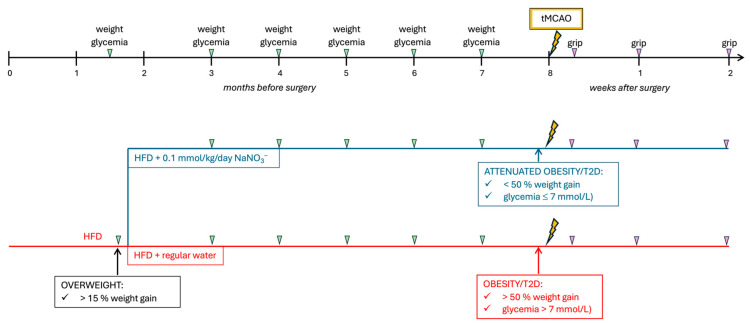
Experimental design of the study. Mice were kept on high-fat diet (HFD) for 6 weeks to induce overweight (>15% weight gain compared with lean, age-matched controls). Then animals were randomly allocated to two groups: one group received HFD and 0.1 mmol/kg/day dietary nitrate (NaNO_3_^−^) in drinking water and one group was continued on HFD and regular water. After 24 weeks, when obesity and diabetic features (T2D) were present in the group on HFD and regular water, all animals were subjected to transient middle cerebral artery occlusion (tMCAO) and recovery was tracked for 2 weeks thereafter. Green triangles indicate weight and glycemia check, pink triangles indicate grip strength test.

Starting at 4 weeks of age, the mice were exposed to a high-fat diet (HFD; 60% energy from fat). The animals became overweight after 6 weeks of the HFD (defined as 15–25% weight gain compared with lean, age-matched mice). Thereafter, they were randomized into two groups: HFD and 0.1 mmol/kg/day NaNO_3_^−^ (NO_3_^−^, *n* = 10) in drinking water and HFD and regular water (HFD, *n* = 10). Dietary nitrate was administered as described previously [21]. Briefly, NaNO_3_^−^ was added to the drinking water at a concentration of 85 mg/L (1 mM). This resulted in a daily intake of 0.1/mmol/kg, a dose that corresponds to a daily intake of 100–300 g of a nitrate-rich food in humans and has proven efficacious in reversing metabolic syndrome in previous experiments [21,36]. Body weight and fasting glucose were monitored every 4 weeks until the HFD group developed obesity (defined as >50% weight gain compared with lean, age-matched mice) and hyperglycemia (defined as fasting glucose >7 mmol/L). Then, insulin resistance was assessed via an insulin and glucose tolerance test, confirming the development of obesity and diabetes in the HFD group after 24 weeks of the HFD. Then, the mice were subjected to transient middle cerebral artery occlusion (tMCAO) to induce stroke, and functional recovery was tracked using the grip strength test (see below). An overview of the experimental design can be found in Figure 1.

### 2.4. Metabolic Assessments

#### 2.4.1. Fasting Glycemia

Fasting glycemia was measured every 4 weeks during NO_3_^−^ supplementation via a tail tip puncture and a glucometer after overnight (ON) fasting.

#### 2.4.2. Insulin Tolerance Test (ITT)

An ITT was performed after 24 weeks of NO_3_^−^ supplementation. Briefly, the mice were fasted for 2 h and baseline glycemia was measured via tail tip puncture and a glucometer. Then, animals were injected with 0.5 U/kg human insulin intra-peritoneally (i.p.) and glycemia was checked at 15, 30, 45, 60, 75, and 90 min after injection. The percentage of baseline glycemia was calculated and the area under the curve was used for statistical analysis.

#### 2.4.3. Glucose Tolerance Test (GTT)

A GTT was performed after 24 weeks of NO_3_^−^ supplementation. Briefly, the mice were fasted ON. Then, baseline glycemia was measured and mice were injected i.p. with 1 g/kg glucose. Glycemia was monitored at 15, 30, 60, 90, and 120 min after injection. The percentage change from baseline glycemia was calculated and the area under the curve was used for statistical analysis.

### 2.5. Transient Middle Cerebral Artery Occlusion

Stroke was induced by tMCAO using the intraluminal filament technique as described previously [37,38]. Briefly, the mice were anesthetized by the inhalation of 3% isoflurane and, throughout surgery, anesthesia was maintained by 1.5% isoflurane. Using a heated pad with feedback from a thermometer, the body temperature of the animals was kept at 37–38 °C. The left external (ECA) and internal (ICA) carotid arteries were exposed and a 7–0 silicone-coated monofilament (total diameter 0.17–0.18 mm) was inserted into the ICA until the origin of the MCA was blocked. The occluding filament was removed after 35 min. Cerebral blood flow in the vicinity of the MCA was monitored by a Laser Doppler Blood Flow Monitor (Moor Instruments Ltd., Axminster, UK), and no differences between the groups were observed. Stroke induction was considered unsuccessful when the occluding filament could not be advanced within the internal carotid artery beyond 7–8 mm from the carotid bifurcation, or if the mice lacked symptoms of neurological impairment based on the neurological severity score [39]. After surgery, all mice were given an analgesic (Carprofen, 5 mg/kg) and soft food. After tMCAO, all mice were switched to normal chow, to mimic the clinical setting of a post-stroke balanced diet. Then, two mice in the HFD-group and one mouse in the NO_3_^−^ group were euthanized shortly after surgery because the humane endpoint was reached (HFD *n* = 8, NO_3_^−^ *n* = 9).

### 2.6. Behavioral Assessment

To assess functional recovery after stroke, forelimb grip strength was tested as previously described [38,40]. Briefly, the mice were held firmly by the body and allowed to grasp the grid with the paretic (right) forepaw. Hereafter, they were dragged backwards until their grip was broken. Grip strength was measured using a grip strength meter (Harvard apparatus, Holliston, MA, USA) at 3 days and 1–2 weeks after tMCAO. Ten trials were performed by an experimenter blinded to the groups, and the average of the two highest values was used for statistical analysis.

### 2.7. Tissue Collection

After a 4 h fast, the animals were anesthetized using an overdose of sodium pentobarbital. Hereafter, cardiac puncture was performed to collect blood, and then the mice were perfused transcardially using PBS followed by a 4% ice-cold paraformaldehyde (PFA) solution. Brains were harvested and stored ON in 4% PFA at 4 °C. After 24 h of PFA fixation, brains were transferred to a solution of PBS and 25% sucrose and stored at 4 °C until they sank. Then, 30 μm thick coronal sections were cut using a sliding microtome, and sections were stored at −20 °C in anti-freeze solution until further analysis.

### 2.8. Immunohistochemistry (IHC)

To stain the brain sections, the free-floating staining method was used. Briefly, sections were washed in PBS to remove anti-freeze solution. The sections were then incubated in a solution of 3% H_2_O_2_, 10% MeOH, and PBS, for visualization with 3′-3 diaminobenzidine (DAB). For immunofluorescent staining with Ki67 and double-cortin (DCX), sections were then incubated for 15 min in citric acid (pH = 6.0) at 95 °C for antigen retrieval. For immunofluorescent staining with aminopeptidase N (CD13), podocalyxin (PDXL), a blocking step was performed with a 1 h incubation in PBS supplemented with 0.25% Triton-X-100 and 5% serum at room temperature (RT). Hereafter, the sections were incubated ON at 4 °C in a PBS solution supplemented with a primary antibody, 3–5% normal horse or normal donkey serum, and 0.25% Triton-X-100. For NeuN, the sections were incubated in a primary antibody solution for 48 h. The following primary antibodies were used: NeuN (1:500; RRID: AB_2298772), Ki67 (1:300; RRID: AB_443209), DCX (1:200; RRID: AB_10610966), Iba1 (1:1000; RRID: AB_2220422), CD206 (1:200; RRID: AB_2063012), CD68 (1:2000, RRID: AB_10975465), PDXL (1:200; RRID: AB_354858), and CD13 (1:200; RRID: AB_323691). After incubation with a primary antibody, the sections were washed and incubated with a secondary antibody solution consisting of PBS supplemented with a secondary antibody, 3–5% normal horse or normal donkey serum, and 0.25% Triton-X-100 for 2 h at RT. The following secondary antibodies were used: biotinylated horse anti-mouse (1:200; RRID: AB_2313581), biotinylated horse anti-goat (1:200; RRID: AB_2336123), Alexa-488 conjugated horse anti-rabbit (1:200; RRID: AB_2336403), Alexa-594 conjugated horse anti-mouse (1:200; RRID: AB_2336412), Alexa-594 conjugated donkey anti-goat (1:500; RRID: AB_2340432), and Cy5 conjugated donkey anti-rat (1:500; RRID: AB_2340671). A 1 h incubation with avidin–biotin complex was next performed for the stainings for DAB visualization according to the manufacturer’s instructions (Vectastain Elite ABC kit, Vector Laboratories, Newark, CA, USA), followed by DAB visualization. All quantifications of the IHC stainings were performed by experimenters blinded for the groups.

### 2.9. Analysis

#### 2.9.1. Quantification of Infarct Size

NeuN-labeled sections were displayed on a computer monitor using a 1.25× lens. Infarct size was determined using all serial sections containing visual ischemic damage. The volume of the contralateral, non-injured hemisphere and of the intact portion of the ipsilateral, injured hemisphere were measured using the Cavalieri Estimator probe (StereoInvestigator, MBF Bioscience, Williston, VT, USA) [41]. Then, the infarct size was calculated by subtracting the ipsilateral volume from the contralateral volume, to adjust for stroke-induced tissue shrinkage. Animals with extensive cortical damage (three animals in the NO_3_^−^ group) were excluded for analysis.

#### 2.9.2. Quantification of Stroke-Induced Neural Stem Cell Proliferation (Ki67) and Early Neurogenesis (DCX)

Animals with extensive cortical damage (three animals in the NO_3_^−^ group) were excluded from these analyses. Manual counting of Ki67 in the subventricular zone (SVZ) and of DCX in striatum was performed as described previously [42]. Briefly, three coronal sections were manually counted using the Olympus BX40 microscope. The first section was selected based on its anatomical location along the rostral–caudal axis (approximately 1 mm from the Bregma). The second and third sections were 300 and 600 μm caudal from the first section. The number of Ki67^+^ cells in the SVZ and of DCX^+^ cells in striatum was manually counted in all three sections using a dry 40× lens. All counts were performed by experimenters blinded to the treatment groups. The sum of the counts in all three sections was used for statistical analysis.

#### 2.9.3. Quantification of Neuroinflammation

The Fiji open-source software [43] was used to evaluate Iba1 immunoreactivity as described previously [42]. Animals with extensive cortical damage (three animals in the NO_3_^−^ group) were excluded from these analyses. Briefly, three coronal sections were included in the analysis. The first section was selected based on its anatomical location along the rostral–caudal axis (approximately 1 mm from the Bregma). The second and third sections were 300 and 600 μm caudal from the first section. Images of Iba1 staining in striatum were acquired at 20× using the Olympus BX40 microscope. For each contralateral, undamaged hemisphere, one representative image of each section was acquired. For the ipsilateral, injured hemisphere, three images per section spanning >90% of the whole striatum were acquired, accounting for nine images in total for each animal. Then, the images were converted into grayscale (8-bit) mode, and a threshold was determined based on the lowest Iba1 immunoreactivity in the contralateral striatum of the HFD group. The Iba1^+^ area was then measured and expressed as a percentage of the total area that was analyzed.

The number of CD68^+^ cells in ipsilateral striatum and CD206^+^ cells in the peri-infarct area was counted manually in three coronal sections. Animals with extensive cortical damage (three animals in the NO_3_^−^ group) were excluded from these analyses. The first section was selected based on its anatomical location along the rostral–caudal axis (approximately 1 mm from the Bregma). The second and third sections were 300 and 600 μm caudal from the first section. For CD68^+^ quantification, positive cells were counted manually in the ipsilateral striatum using a 60× dry lens. The sum of the counts in three sections was used for statistical analysis. For the quantification of CD206 in the peri-infarct area, first the area of interest was delineated at 4× magnification and subsequently, cells were manually quantified using a 60× dry lens in the delineated area. Then, the number of positive cells per area was calculated and used for statistical analysis.

#### 2.9.4. Quantification of Neovascularization

Animals with extensive cortical damage (three animals in the NO_3_^−^ group) were excluded from these analyses. Neovascularization was quantified using the open-source software Fiji [43]. Confocal images were acquired with a Leica DMi8 confocal microscope. For each animal, one brain section was selected, and one to two images were captured per section at 20× magnification in the peri-infarct area. This region was identified by comparing the immunofluorescence signal to previously acquired reference images showing the ischemic core based on NeuN staining. Image dimensions were 775 μm × 775 μm, with a z-stack depth of 10 μm and a step size of 0.5 μm. The same acquisition settings were used for all images. Vascular parameters were quantified on maximum intensity projections of thresholded images using Fiji’s area fraction measurement tool. The area density was expressed as the percentage of PDXL and CD13 of the total image area. Pericyte coverage was calculated by measuring the area of colocalized CD13 and PDXL signals and normalizing it to the total PDXL-positive area within the same image.

For vessel length quantification, the maximum projected images were thresholded and skeletonized. The resulting skeletons were analyzed using the AnalyzeSkeleton plugin [44], as previously described [45]. To measure vascular diameter, we adapted the Vessel Analysis plugin [46,47]. Briefly, following maximum projection and thresholding, a Euclidean distance map was generated to indicate the distance of each pixel to the nearest background pixel. Simultaneously, skeletonized versions of the images were created. The multiplication of the distance map with the skeletonized images produced skeletons containing vessel thickness information, from which the average vessel diameter was calculated. All image analyses were scripted and automated using the ImageJ Macro language (NIH, Bethesda, MD, USA) to minimize human error and bias. Macro scripts are publicly available at: https://github.com/carbui/image-analysis/ (accessed on 1 May 2023).

### 2.10. Statistical Analysis

GraphPad prism version 10 (10.2.3 (347)) was used for statistical analysis. Data was checked for statistical outliers using the ROUT method, and for normality using the Shapiro–Wilk test. For body weight, weight change, fasting glucose, and grip over time, Ki67, DCX, Iba1, CD206, PDXL, CD13, CD13/PDXL ratio, vessel length, and vessel diameter were analyzed using a repeated measures two-way ANOVA with the two-stage linear set-up procedure of Benjamini, Krieger, and Yekutieli. For the AUC of ITT and grip, fasting glucose, stroke volume, and CD68 quantification an unpaired *t*-test was used. For the AUC of GTT, Welch’s t-test was used. All data are expressed as mean ± SD and results were considered significant when *p* < 0.05.

## 3. Results

### 3.1. Sustained NO_3_^−^ Supplementation in Overweight Mice Attenuates the Development of Obesity and Hyperglycemia and Improves Stroke Recovery

**Figure 2:**
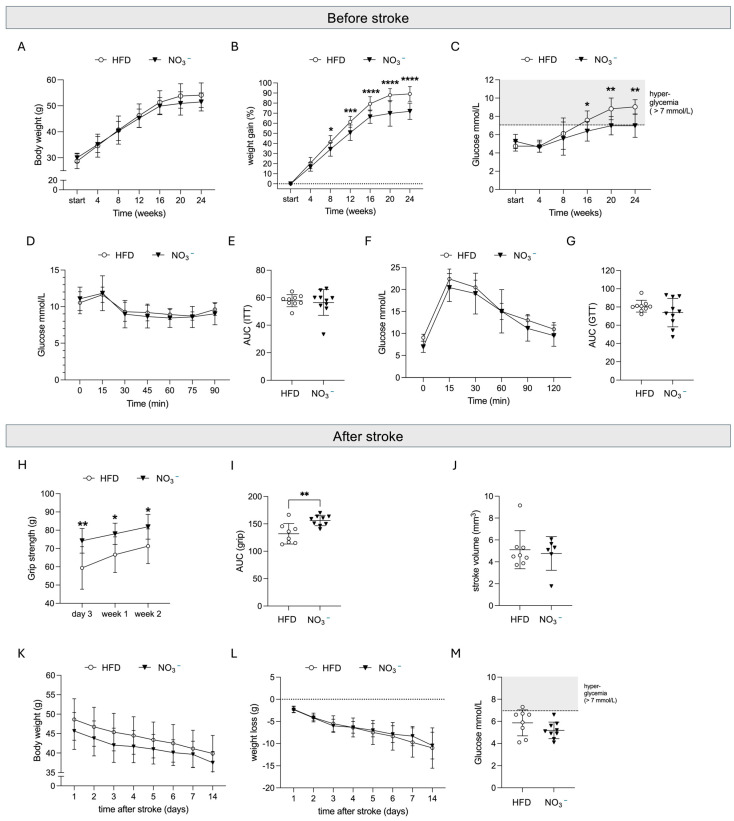
Effect of prolonged NO_3_^−^ supplementation on pre-stroke metabolism and stroke outcome. The effect of 24 weeks of NO_3_^−^ on (**A**) body weight (in grams), (**B**) weight gain in %, (**C**) fasting glucose over time, and (**D**,**E**) insulin sensitivity, measured with an insulin tolerance test (plotted curve in (**D**), area under the curve in (**E**)), and a (**F**,**G**) glucose tolerance test (plotted curve in (**F**), area under the curve in (**G**)). (**H**,**I**) Forepaw grip strength, as plotted curve (**H**) and area under the curve (**I**). (**J**) Stroke volume. (**K**) Body weight (grams), (**L**) weight loss (grams), and (**M**) fasting glucose at two weeks after stroke. Data are presented as mean ± SD. Statistical significance was calculated using repeated measures two-way ANOVA, with the two-stage linear set-up procedure of Benjamini, Krieger, and Yekutieli in (**A**–**C**,**H**,**K**,**L**), unpaired t-test in (**E**,**I**,**J**,**M**), and Welch’s t-test in (**G**). Results were considered significant when *p* < 0.05. * depicts a significant difference between HFD and NO_3_^−^ with * denoting *p* < 0.05, ** denoting *p* < 0.01, *** denoting *p* < 0.001, and **** denoting *p* < 0.0001. HFD = high-fat diet, NO_3_^−^ = dietary nitrate. Sample size: (**A**–**G**) *n* = 10 per group, (**H**,**I**,**K**–**M**) HFD *n* = 8, NO_3_^−^ *n* = 9, (J) HFD *n* = 8, NO_3_^−^ *n* = 6.

Six weeks of HFD feeding induced an overweight condition compared with age-matched controls fed a standard diet (Supplementary Material Figure S1). Although NO_3_^−^ supplementation did not prevent the development of obesity (Figure 2A), a 15% attenuation in weight gain was observed in the NO_3_^−^ group (Figure 2B). Importantly, HFD-mice developed hyperglycemia during the duration of the study, reaching fasting glycemia levels of 9 mmol/L after 24 weeks (Figure 2C). On the contrary, fasting glycemia remained significantly lower in NO_3_^−^-treated mice and below the diabetic threshold of 7 mmol/L (Figure 2C). No differences between the groups were observed when performing insulin (Figure 2D,E) and glucose tolerance tests (Figure 2F,G), indicating that long-term NO_3_^−^ supplementation did not improve insulin sensitivity.

These results indicate that although long-term NO_3_^−^ supplementation did not prevent the occurrence of insulin resistance and obesity in overweight mice, it prevented the occurrence of hyperglycemia and slightly reduced weight gain, even under continuous HFD feeding.

After stroke, grip strength was significantly greater in the NO_3_^−^ group compared with the HFD mice (Figure 2H,I) despite no difference in stroke volume between the groups (Figure 2J). In accordance with previous studies [38,42], all mice lost weight after tMCAO, with no differences in the degree of weight loss between the groups (Figure 2K,L). This rapid post-stroke weight loss resulted in attenuated hyperglycemia in both groups at 2 weeks after stroke (Figure 2M).

Taken together, these data show that long-term supplementation with NO_3_^−^ significantly improves functional recovery after stroke without affecting stroke size. Moreover, our results indicate that this improved stroke recovery was associated with an attenuation of HFD-induced weight gain and with the prevention of hyperglycemia before stroke.

### 3.2. Improved Stroke Outcome in NO_3_^−^ Mice Was Not Associated with Increased Stroke-Induced Early Neurogenesis

**Figure 3:**
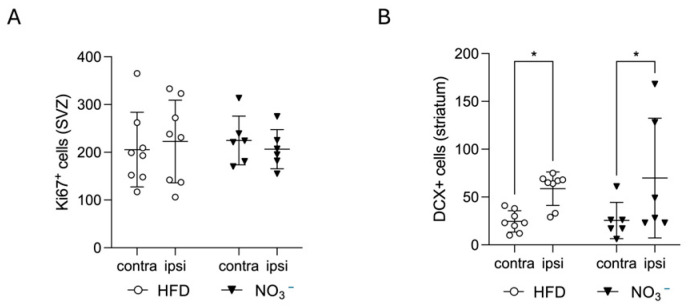

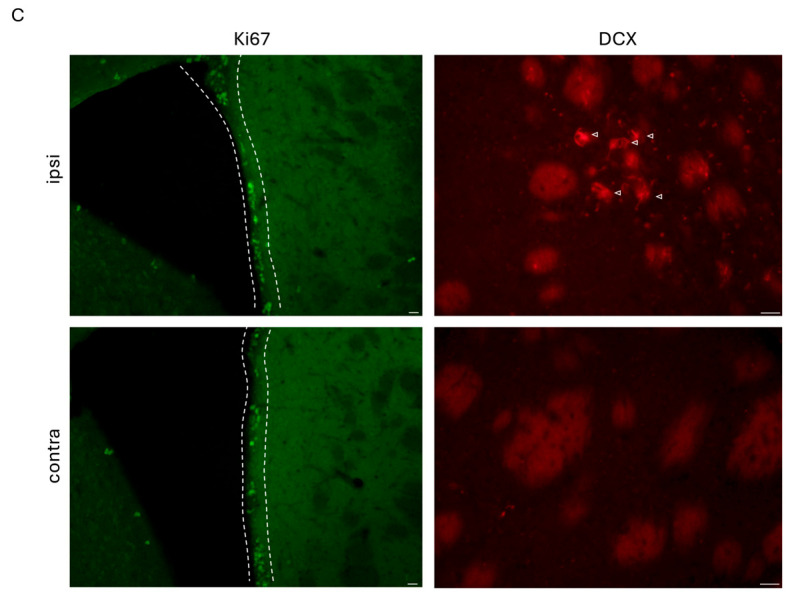
Effect of long-term NO_3_^−^ supplementation on post-stroke neurogenesis. (**A**) Number of Ki67^+^ cells in subventricular zone (SVZ) and (**B**) number of DCX^+^ cells in striatum. (**C**) Representative images of Ki67 (left) and DCX positive staining. The white dotted line on the Ki67 images delineates subventricular zone, white arrows on the DCX images indicate DCX^+^ cells. Scale bar = 20 μm. Data are presented as mean ± SD. Statistical significance was calculated using repeated measures two-way ANOVA, with the two-stage linear set-up procedure of Benjamini, Krieger, and Yekutieli. Results were considered statistically significant when *p* < 0.05 and * denotes *p* < 0.05. HFD = high-fat diet, NO_3_^−^ = dietary nitrate, SVZ = subventricular zone. Sample size: HFD = 8, NO_3_^−^ = 6.

NO_3_^−^ has been shown to increase adult neurogenesis [48]. Since stroke-induced neurogenesis has been associated with improved stroke recovery [49], we next assessed the effect of long-term NO_3_^−^ supplementation on this cellular process. The number of Ki67^+^ cells in the SVZ was not different between the groups, showing that stroke-induced neural stem cell proliferation was not altered by NO_3_^−^ supplementation (Figure 3A). Moreover, the amount of DCX^+^ neuroblasts in the ipsilateral striatum was significantly higher compared with the contralateral striatum in both groups (Figure 3B). However, the number of DCX^+^ cells was not different between the groups (Figure 3B). These results show that improved recovery in the NO_3_^−^ group was not associated with differences in stroke-induced early neurogenesis.

### 3.3. Improved Stroke Outcome in NO_3_^−^ Mice Was Associated with Decreased Post-Stroke Inflammation

**Figure 4:**
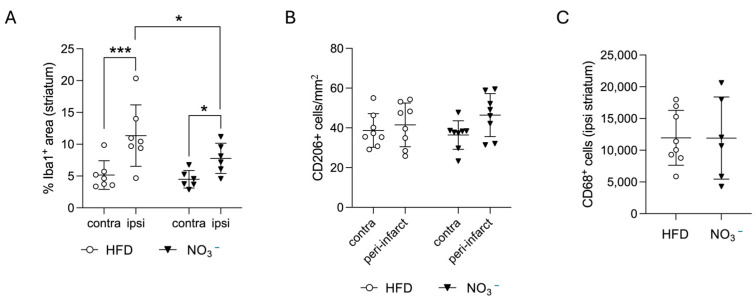

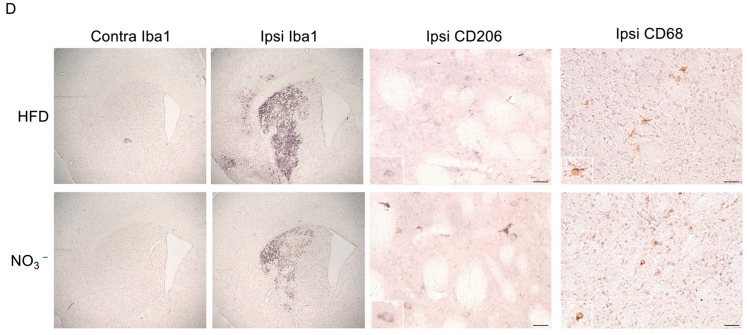
Effect of long-term NO_3_^−^ supplementation on post-stroke neuroinflammation. (**A**) Striatal Iba-1 expression, % positive area. (**B**) Number of CD206^+^ cells in contralateral striatum and ipsilateral peri-infarct per mm^2^, average of three sections. (**C**) Number of CD68^+^ cells in ipsilateral striatum. (**D**) Representative images of Iba1, CD206, and CD68 staining. Scale bar = 20 μm. Data are presented as mean ± SD. Statistical significance was calculated using repeated measures two-way ANOVA, with the two-stage linear set-up procedure of Benjamini, Krieger, and Yekutieli in (**A**,**B**), and unpaired t-test in (**C**). Results were considered statistically significant when *p* < 0.05, with * denoting *p* < 0.05, and *** denoting *p* < 0.001. HFD = high-fat diet, NO_3_^−^ = dietary nitrate. Sample size: HFD = 8, NO_3_^−^ = 6.

Neuroinflammation plays a critical role in stroke recovery but is dysregulated by T2D [50]. Therefore, we investigated whether NO_3_^−^ impacted post-stroke neuroinflammation. We quantified Iba1 immunoreactivity in both the ipsilateral and the contralateral hemisphere to assess microglial infiltration. In accordance with previous studies, we found significantly higher Iba1 expression in the ipsilateral compared with the contralateral hemisphere (Figure 4A). Importantly, the amount of Iba1 in the ipsilateral hemisphere was significantly lower in the NO_3_^−^ group compared with the HFD (Figure 4A,B), indicating a dampened inflammatory response in NO_3_^−^-treated mice.

Next, we characterized whether this post-stroke neuroinflammation resembled either a more proinflammatory M1-type, indicative of persistent inflammation, or a reparative M2-type response. To identify M2-type immune cells, we quantified the number of CD206^+^ cells, a widely recognized representative M2 microglial marker [51,52,53], in both the contralateral hemisphere and the peri-infarct area of the ipsilateral striatum. We found no difference between the groups, neither in the peri-infarct CD206^+^ cells (Figure 4B,D) nor in the number of ipsilateral CD68^+^ cells (Figure 4C,D), indicating that the decreased microglial infiltration was not accompanied by a shift in the balance between M1- and M2-type microglia [54].

Taken together, these results indicate that sustained NO_3_^−^ supplementation improves stroke outcome in association with decreased ipsilateral microglial infiltration, which is indicative of reduced post-stroke neuroinflammation.

### 3.4. Improved Stroke Outcome in NO_3_^−^ Mice Was Not Associated with Improved Post-Stroke Vascularization

**Figure 5:**
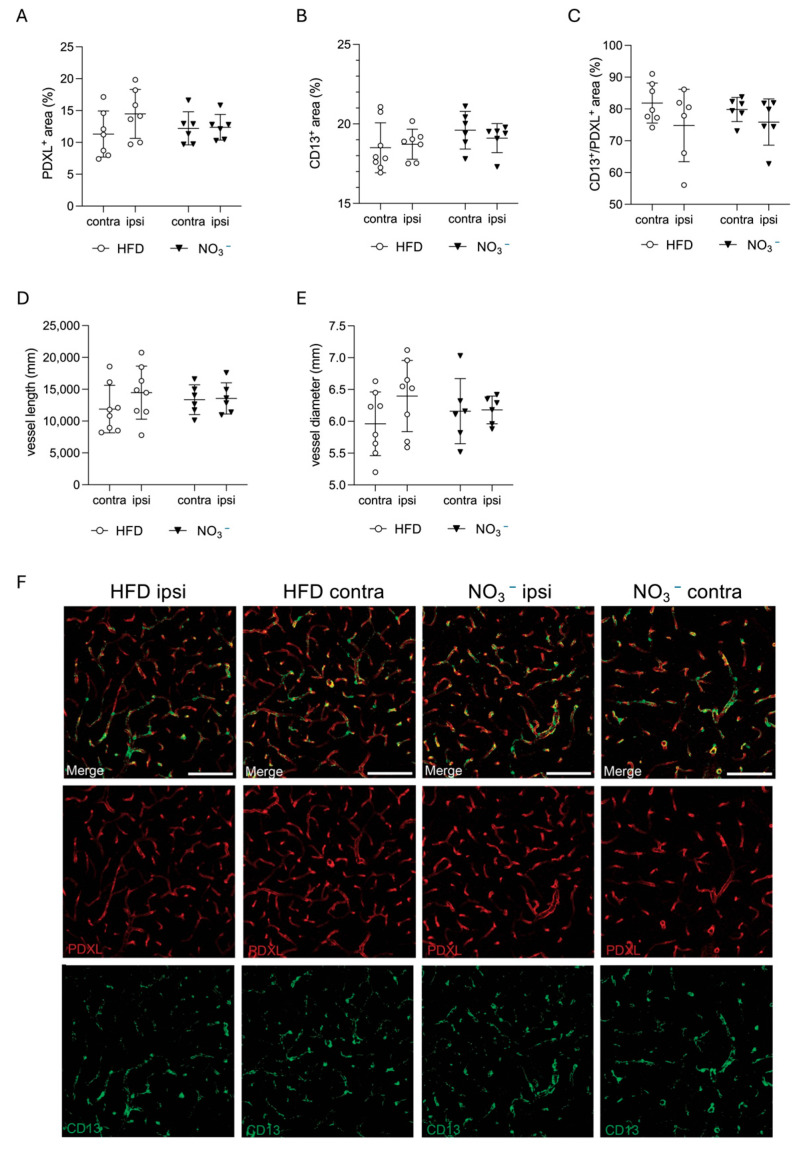
Effect of long-term NO_3_^−^ supplementation on post-stroke neovascularization. Expression of PDXL (**A**) and CD13 (**B**) in peri-infarct area. (**C**) Ratio of CD13/PDXL in peri-infarct area. Total vessel length (**D**) and average vessel diameter (**E**) in peri-infarct area. (**F**) Representative confocal images showing comparable pericyte (CD13, green) and vessel (PDXL, red) area density, pericyte coverage, total vessel length, and average vessel diameter across groups. Scale bar = 100 μm. Data are presented as mean ± SD. Statistical significance was calculated using repeated measures two-way ANOVA, with the two-stage linear set-up procedure of Benjamini, Krieger, and Yekutieli. Results were considered statistically significant when *p* < 0.05. HFD = high-fat diet, NO_3_^−^ = dietary nitrate. Sample size: HFD = 8, NO_3_^−^ = 6.

The beneficial effect of NO_3_^−^ on vascularization is well known [55,56,57]. Since post-stroke neovascularization plays an important role in stroke recovery, and diabetes hampers functional angiogenesis [58,59], we next assessed the effect of NO_3_^−^ supplementation on post-stroke vascularization in the peri-infarct area. We found no difference between the groups in PDXL staining density, indicating that NO_3_^−^ did not alter the overall vessel area (Figure 5A). Moreover, there was no difference in the CD13^+^ area between the groups (Figure 5B), nor in the ratio between CD13^+^/PDXL^+^ (Figure 5C), suggesting that NO_3_^−^ did not alter the number of pericytes, or the pericyte coverage of vessels after stroke. In addition, neither the total vessel length (Figure 5D) nor the average vessel diameter (Figure 5E) in the peri-infarct area was different between the groups.

Taken together, these data indicate that NO^3−^ supplementation did not affect post-stroke neovascularization.

## 4. Discussion

In this study, we showed that sustained NO_3_^−^ supplementation in overweight mice attenuates weight gain, and prevents the occurrence of hyperglycemia, even during ongoing HFD feeding. Importantly, these metabolic effects may contribute to enhanced functional recovery after stroke. Moreover, we showed that improved stroke recovery by NO_3_^−^ was associated with reduced neuroinflammation, but not with improved post-stroke neovascularization or increased neurogenesis.

Being overweight significantly increases the risk of stroke and worsens stroke outcome, predominantly by increasing the likelihood of obesity and T2D, both of which are known to raise the risk of stroke and severe post-stroke disability [60,61,62,63]. However, since being overweight itself is not classified as a disease, pharmacological interventions are hard to justify in this group and current recommendations to halt the progression from being overweight to obesity and T2D predominantly consist of generic lifestyle changes that often lack long-term adherence.

Dietary NO_3_^−^ has been proven to be an effective and feasible strategy to prevent obesity and metabolic complications [22,64,65]. In the current study, under continuous HFD feeding, dietary NO_3_^−^ did not prevent the occurrence of obesity in overweight mice. However, it did slow weight gain by 15% and significantly improved functional recovery after stroke. Interestingly, a 5–10% body weight reduction has proven sufficient to improve metabolic health in obesity [9,66,67] and decrease the risk of cardiovascular complications [68]. This suggests that even a modest effect in reducing weight gain, as shown in this study, could be important to improve stroke recovery. Moreover, the composition of HFDs used in murine studies is typically more extreme than the dietary patterns observed in humans with obesity [69,70,71]. Considering that NO_3_^−^ is a dietary supplement and not a medication, it is notable that even under concomitant exposure to an aggressive HFD, weight gain was still attenuated.

In the present study, NO_3_^−^ did not prevent insulin resistance. Since insulin sensitivity closely correlates with weight gain, and both groups developed obesity after 24 weeks of the HFD, this could explain the lack of difference in insulin sensitivity [72]. However, even though the mice were insulin-resistant, NO_3_^−^ did prevent hyperglycemia entirely. Indeed, several studies show insulin-independent mechanisms by which dietary nitrate lowers glycemia, for instance through the stimulation of AMPK signaling and via enhancing GLUT4 expression [73,74,75], thereby effectively enhancing glucose uptake and lowering glycemia. Given the well-established link between hyperglycemia and poor stroke outcome [76,77,78], it is conceivable that the improved stroke recovery by NO_3_^−^ in our study was mainly mediated by the prevention of hyperglycemia.

In contrast to previous reports [31,32,79], we did not observe any acute neuroprotective effects of NO_3_^−^ supplementation, as infarct size was not different between the groups. NO_3_^−^ was given in the drinking water, which limited the control over its intake relative to the time of stroke induction. Given the relatively short half-life of NO_3_^−^, especially in mice [80], it is likely that we did not observe any neuroprotective effects simply because the NO_3_^−^ concentrations were insufficient at the time of stroke to mediate neuroprotection. Consequently, this suggests that the metabolic effects of NO_3_^−^ before stroke were likely key contributors to the improved stroke recovery observed. How improved stroke recovery can be achieved in the absence of changes in stroke volume remains unclear but may involve enhanced neuroplasticity during the recovery phase [81,82].

In the present study, NO_3_^−^ administration was initiated at the onset of being overweight and continued through the post-stroke period. While this experimental design reflects a clinically relevant scenario, it limits the ability to discern whether the improved stroke recovery observed with NO_3_^−^ was due to pre-stroke, post-stroke, or combined effects. However, given the absence of differences in infarct size and post-stroke metabolism between the groups, it is tempting to speculate that the beneficial pre-stroke effects of NO_3_^−^ could have played a causal role in the improved recovery seen in the NO_3_^−^ group. A follow-up study, restricting NO_3_^−^ administration to the pre-stroke time frame would help clarify the specific metabolic mechanisms underlying this effect.

Neuroinflammation plays a crucial role in stroke outcome [83], and diabetes has been shown to exacerbate post-stroke neuroinflammation, thereby worsening stroke outcome [50]. In this study, we demonstrated that NO_3_^−^ reduced microglial infiltration, indicating reduced post-stroke neuroinflammation which has been associated with improved post-stroke recovery [84]. Indeed, microglia are key modulators of neuroplasticity, both under homeostatic conditions and in the post-stroke brain, when effective neuroplasticity is essential for the formation of new neuronal connections [84,85,86]. Our data suggests that the improved stroke recovery observed with NO_3_^−^ supplementation may be mediated by enhanced neuroplasticity secondary to reduced neuroinflammation.

Post-stroke neovascularization is impaired in the context of T2D and is associated with poorer stroke recovery [59]. Nitrates, commonly used as vasodilators in the treatment and prevention of angina pectoris, have demonstrated various vascular benefits across different diseases [55,56]. However, their role in post-stroke cerebral vascularization remains poorly understood. In our study, NO_3_^−^ supplementation did not alter the vascular parameters following stroke. Nevertheless, potential effects on the cerebral vasculature cannot be entirely ruled out. Hypertension is a well-established factor that worsens stroke outcome [87], and NO_3_^−^ can reduce hypertension in the brain after ischemic injury [26,32,34]. In the present study, blood pressure was not monitored, leaving the potential associations of improved stroke recovery by NO_3_^−^ with lowered blood pressure unexplored.

Stroke-induced neurogenesis has been associated with improved stroke recovery, and some studies indicate positive effects of NO_3_^−^ on neurogenesis [48,49]. However, we observed no effects of NO_3_^−^ on post-stroke neurogenesis, at least at the time points assessed.

## 5. Conclusions

In conclusion, we demonstrate for the first time the efficacy of a previously unexplored strategy to improve stroke outcome using NO_3_^−^ supplementation in overweight mice, even during continuous HFD feeding. If clinically validated, this prophylactic approach could benefit the population of individuals who are overweight and thus at a higher risk for the development of obesity and T2D and subsequently for stroke and severe post-stroke disability. By receiving NO_3_^−^, such individuals may experience improved metabolic function, even in the presence of ongoing unhealthy dietary habits. This metabolic improvement could prove sufficient to lower their risk for poor stroke prognosis.

## Data Availability

The data that support the findings of this study are not openly available due to reasons of sensitivity and are available from the corresponding author upon reasonable request. Data are located in a controlled access data storage at Karolinska Institutet.

## Supplementary Materials

The following supporting information can be downloaded at: https://www.mdpi.com/article/10.3390/nu17152434/s1. Supplementary Figure S1: 6 weeks of high fat feeding induced overweight. (a) Body weight of mice kept on high-fat diet for 6 weeks compared to body weights of lean, age-matched controls. Mean body weight SD: 23.9 grams, mean body weight HFD: 29.2 grams. On average, animals in the HFD group had gained 17.2% more weight compared to lean, age-matched controls, confirming the establishment of overweight in the HFD-group. Data are depicted as mean ± SD. Statistical analysis was performed using unpaired t-test and p < 0.05 was considered significant. N = 20 per group.
