# Supplementary material for: Prevention of Metabolic Impairment by Dietary Nitrate in Overweight Male Mice Improves Stroke Outcome
Source: Nutrients. 2025 Jul 25;17(15):2434. doi: 10.3390/nu17152434 (PMC12348083; doi:10.3390/nu17152434)
Supplement: Supplementary file 1 [file nutrients-17-02434-s001.zip › nutrients-3727020-supplementary.pdf]

Prevention of Metabolic Impairment by Dietary Nitrate in Overweight Male Mice Improves Stroke Outcome: Supplementary data

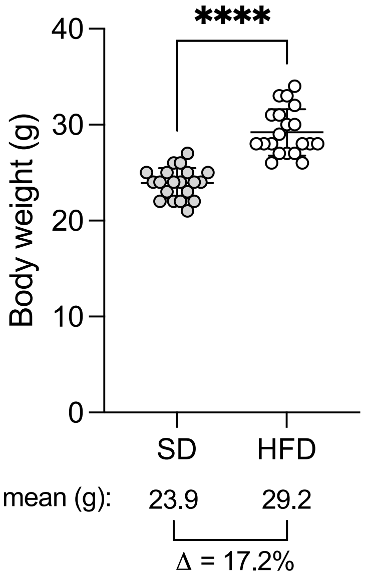

Supplementary Figure S1. 6 weeks of high fat feeding induced overweight. (a) Body weight of mice kept on high-fat diet for 6 weeks compared to body weights of lean, age-matched controls. Mean body weight SD: 23.9 grams, mean body weight HFD: 29.2 grams. On average, animals in the HFD group had gained 17.2% more weight compared to lean, age-matched controls, confirming the establishment of overweight in the HFD-group. Data are depicted as mean ± SD. Statistical analysis was performed using unpaired t-test and $p < 0.05$ was considered significant. N = 20 per group.
